# A case study: impact of target surface mesh size and mesh quality on volume-to-surface registration performance in hepatic soft tissue navigation

**DOI:** 10.1007/s11548-020-02123-0

**Published:** 2020-03-27

**Authors:** Georges Hattab, Carina Riediger, Juergen Weitz, Stefanie Speidel

**Affiliations:** 1 Division of Translational Surgical Oncology, National Center for Tumor Diseases (NCT), 01307 Dresden, Germany; 2 Department of Visceral, Thoracic and Vascular Surgery, University Hospital Carl Gustav Carus, 01307 Dresden, Germany

**Keywords:** Soft tissue, Deformation, Surface, Registration, Evaluation

## Abstract

**Purpose:**

Soft tissue deformation severely impacts the registration of pre- and intra-operative image data during computer-assisted navigation in laparoscopic liver surgery. However, quantifying the impact of target surface size, surface orientation, and mesh quality on non-rigid registration performance remains an open research question. This paper aims to uncover how these affect volume-to-surface registration performance.

**Methods:**

To find such evidence, we design three experiments that are evaluated using a three-step pipeline: (1) volume-to-surface registration using the physics-based shape matching method or PBSM, (2) voxelization of the deformed surface to a $1024^3$ voxel grid, and (3) computation of similarity (e.g., mutual information), distance (i.e., Hausdorff distance), and classical metrics (i.e., mean squared error or MSE).

**Results:**

Using the Hausdorff distance, we report a statistical significance for the different partial surfaces. We found that removing non-manifold geometry and noise improved registration performance, and a target surface size of only 16.5% was necessary.

**Conclusion:**

By investigating three different factors and improving registration results, we defined a generalizable evaluation pipeline and automatic post-processing strategies that were deemed helpful. All source code, reference data, models, and evaluation results are openly available for download: https://github.com/ghattab/EvalPBSM/.

## Introduction

In laparoscopic liver surgery, haptic perception is missing and it is more difficult to localize a tumor. Displaying information about target or risk structures such as the tumor or intra-hepatic vessels and bile ducts can be very helpful. Indeed, providing such information in navigation, i.e., intra-operative image guidance, could assist the surgeon during the intervention. This is possible by means of visualization, for example by using stereo-endoscopy, 3D-laparoscopy, and augmented reality (AR). With this aim, visualization relies on registration to obtain the accurate location of structures and objects of interest. However, the liver may deform under pressure exerted by surgical instruments, characterizing soft tissue deformation. Characteristic hepatic tissue deformations occur either in the first operative steps (upon mobilization of the liver or transection of the liver parenchyma), or during laparoscopy by abnormally high pressure of the capnoperitoneum (12–15 mmHg). The grade of deformation depends on the liver stiffness and the patient-specific liver structure. Indeed, a prerequisite to enable accurate augmentation and navigation, in soft tissue navigation, is taking into account such deformations and aligning or registering the pre- and intra-operative patient data. In hepatic soft tissue navigation, a three-step pipeline is employed  [17, 19, 23]. First, the tumor and the organ of interest are segmented in tomographic patient data, e.g., computed tomography (CT) scan. Second, the intra-operative image data, e.g., from a stereo-endoscope, are employed to reconstruct an intra-operative surface of the visible hepatic tissue. Then, this liver surface is aligned to the preoperative model. The latter refers to an initial registration. Beyond the particular case of the liver, registration may be performed using different methods to achieve surgical navigation: manual [13, 18], point-based [6, 25, 26], calibration-based  [3], volume-based [4, 5], or surface-based registration [9]. Indeed, some projects integrate information from different imaging modalities that are readily available in the operating room. In the operating room, registration can be supported by ultrasound. Third, as the liver tissue deforms under pressure exerted by surgical instruments (i.e., manipulation), or nonlinear forces exerted by breathing motion, or other organs (e.g., the heart), an important task is tracking surface changes and registering them. This aspect integrates soft tissue registration continuously and refers to dynamic registration. It is not addressed in this manuscript. However challenging, it is a prerequisite for real-time navigation systems.

Indeed, intra-operative registration has received a lot of efforts from the community. Most approaches address legitimate questions such as improving the accuracy of registration by presenting novel approaches and validating registration results  [16, 20]. However, the quality of the geometry (e.g., the data noise) and how to improve it has not yet been addressed. A couple of works addressed the factor of visible surface in decreasing increments for two different spatial discretizations: a triangular mesh [16] and a voxel grid [15]. Yet, the impact of noise and the geometry quality on registration results remains open.

As there’s ample related work for intra-operative registration, we solely focus on volume-to-surface methods for non-rigid registration in the particular scope of liver surgery. Our claim is that different factors influence non-rigid registration and lack further investigations. We define three factors: the orientation of the captured surface of interest, the mesh quality of its geometrical representation (i.e., the stitched surface), and the surface mesh size.

In non-rigid registration, an example solution is to create a biomechanical model that considers organ models as finite volume meshes and integrates a-priori knowledge about the mechanical properties of the tissue. The general idea is to solve the boundary value problem while taking into account displacement boundary conditions from the intra-operative surface. In this context, various methods rely on a biomechanical model  [2, 7, 10, 14]. Since our goal is to evaluate the effect of different factors on registration performance, we choose the physics-based shape matching or PBSM method as an example algorithm for volume-to-surface registration. Moreover, it outperforms other related work with reports of mean errors smaller than 1 mm  [7, 14]. Compared to other methods, it describes the non-rigid registration as an electrostatic–elastic problem and relies on the finite element method (FEM) [23]. The PBSM electrically charges the initial volume and slides it into an oppositely charged rigid surface or the target surface. The latter is used for the non-rigid registration and is characteristic of a volume-to-surface registration.

**Fig. 1:**
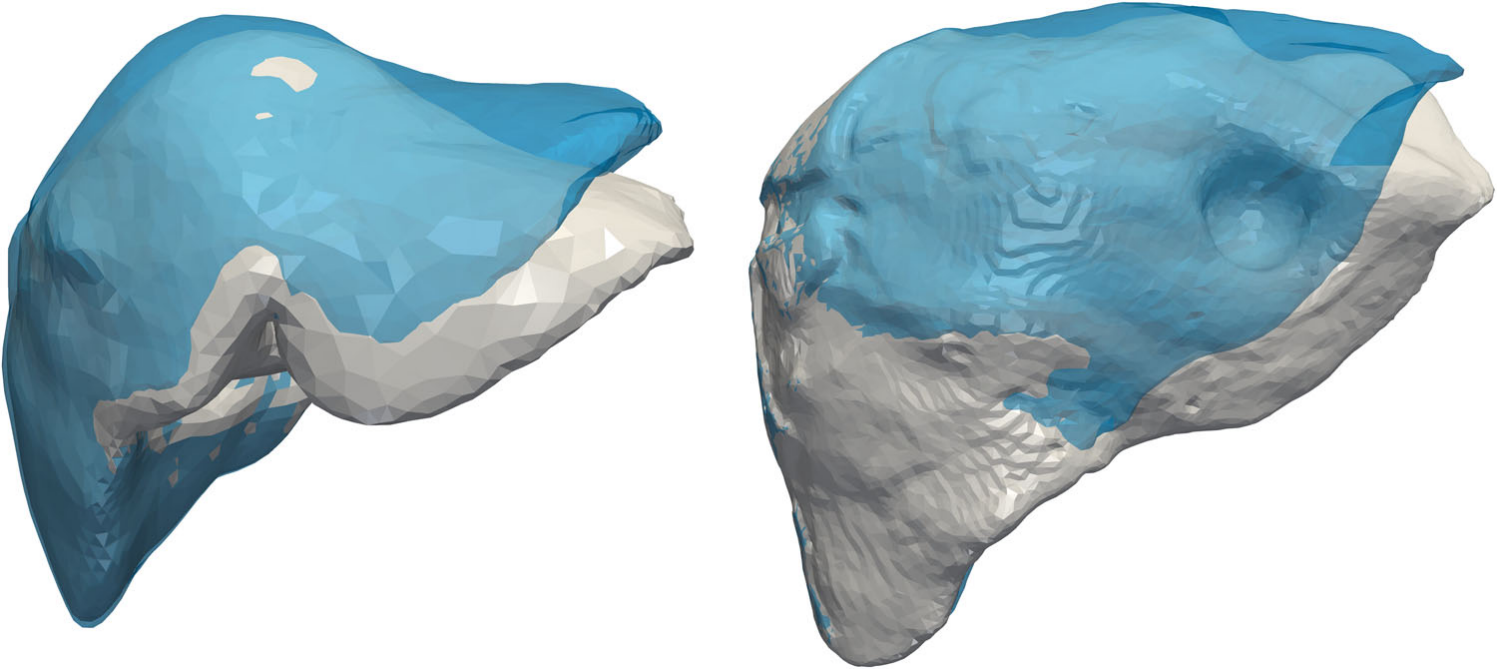
Reference data sets used in the evaluation. The in silico data set (left) and phantom data set (right). The initial surface is depicted (blue) in the same coordinate system as the deformed surface (white)

In an effort to provide baseline data, researchers published experimental phantom data and in silico or simulated data  [23]. Although these data were used to validate ad hoc solutions, the initial surface used for registration was not evaluated in the context of the three aforementioned factors. More generally, these factors remain unaddressed by the related work. Indeed, geometry captures objects from a real environment and represents them digitally by means of discretization. Pre- and intra-operative image data enable us to capture and create surfaces and volumes. However, the geometry quality and how to improve it has not been addressed in a clinical focus. In the case of surface reconstruction, various methods using laparoscopic images exist [8]. Yet, it is until recently that the first reference data were made available via the Stereo Correspondence and Reconstruction of Endoscopic Data Sub-Challenge Part of the Endoscopic Vision Challenge 2019 https://endovissub2019-scared.grand-challenge.org. Moreover, only one related work addressed the question of the required visible surface to obtain good registration results  [15]. However, this work addressed this factor in relation to another spatial discretization of the geometry, i.e., voxel grid. Indeed, the impact of this factor on triangular mesh surfaces and tetrahedral volumes remains an open question.

Motivated by such questions, our goal is to determine the effects of each factor on the registration performance. To this end, we design three specific experiments. First, to characterize the effects of orientation on registration results, we investigate deformation results on both reference phantom data and in silico data  [23] by using directional bisections. Second, to describe the effects of noise and unrealistic geometry, we propose two post-processing strategies to improve the quality of an example stitched target surface and compare results to the unfiltered version. Third and last, to investigate the minimum required target surface size, we conduct a sensitivity analysis by iteratively bisecting the target surface from the laparoscopic point of view and evaluating the registration results.

Herein, we present our evaluation analyses with the following contributions: (a) uncover with statistical significance that target surface size impacts registration performance, (b) discover that a minimum target surface area size of 16.5% is suitable for registration, (c) suggest two automatic strategies that improve target mesh quality, (d) discover that non-manifold geometry and noise (i.e., bad mesh quality) have a negative effect on registration performance, and (e) provide the three-step evaluation pipeline, the required materials, and scripts to conduct future evaluations and analyses.

## Materials and methods

To investigate the formulated questions, we employ reference data from two liver data sets: in silico data set and phantom data set, as shown in Fig. 1.

The in silico data set is a deformed liver by means of a nonlinear biomechanical model. The data comprise initial surface, initial volume, deformed surface, deformed volume, and partial deformed surface.

The phantom data set is a silicone-based liver phantom with patient-specific geometry and realistic elastic properties. The data are obtained from an indentation experiment deforming the liver by using a rigid sphere pushed against the phantom. The deformation is tracked using CT. The data are divided into two, as they are acquired from CT and endoscopic imaging, respectively: initial surface, deformed surface, partial surface of the deformed liver, and partial stitched surface (Endo).

Both data sets are openly available at http://opencas.webarchiv.kit.edu/?q=PhysicsBasedShapeMatching. For comparability and consistency, we evaluate the effect of each factor (orientation, mesh quality, target surface size) on registration performance, by designing three experiments and an evaluation pipeline. We detail each below.

**Fig. 2:**
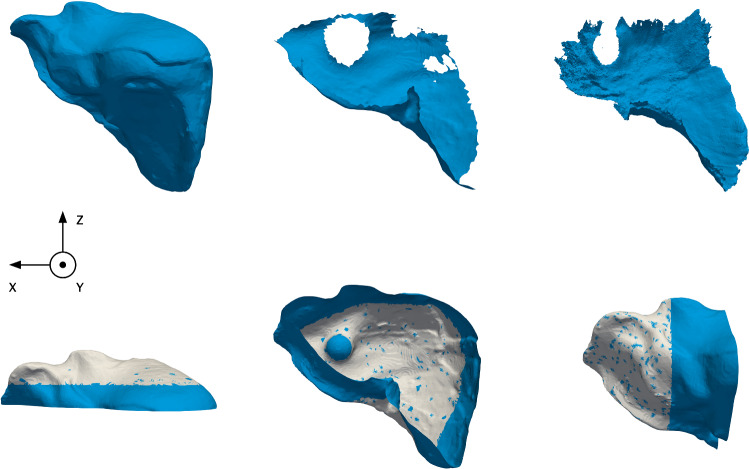
Experiment 1. Phantom liver target surfaces. Each target surface is shown in the *xz* plane and is reported below. Target surfaces are mentioned from left to right and from top to bottom. The top row depicts the available initial non-deformed volume, the partial surface from CT, and the partial or stitched surface from intra-operative endoscopy, respectively. The bottom row depicts the bisected surfaces superimposed for *XY* and *XY*-hemi, *XZ* and *XZ*-hemi, *YZ* and *YZ*-hemi, respectively. Hemi-surfaces are shown in white, while bisected surfaces are shown in blue. For example, in the bottom right corner, the *YZ*-hemi surface (white) superimposes the *XY* surface (blue)

### Evaluation pipeline

We define a three-step pipeline that is target surface size independent:

1. *Volume-to-target surface registration* deforms the volume using the PBSM method. As the PBSM relies on a FEM to slide the electrically charged volume into the target surface, it combines potential and elastic energies. This corresponds to a physics simulation that minimizes the sum of the regularized energy and the potential energy. While the regularized energy of the biomechanical model relies on a material matrix, which contains the Young modulus and the Poisson ratio, the minimization occurs over a number of iterations using discrete time steps [19]. These parameters are set for the in silico and experimental phantom data sets: Poisson ratio ($p=0.4$), Young modulus ($y=10^3$), time step ($dt \in \{0.5, 1\}$), number of iterations ($i \in \{50, 200\}$), and charge ($c \in \{10, 500\}$), respectively. For experiments 2 and 3, the registration parameters of the phantom data set are employed.
2. *Voxelization of the deformed surface* into a $1024^3$ voxels grid [11, 21]. The rational behind using voxelization is to streamline the analysis by making the deformed volumes comparable. Hence, when a meshing algorithm constrains the final element sizes of a volume, the comparison of the two deformed volumes is problematic. The pipeline relieves this, as it is independent of the meshing algorithms used to create a volume. For example, the partial target surface from CT is otherwise incomparable to the stitched surface from endoscopic image data (c.f. phantom data set). The size of the voxel grid is chosen to accommodate as much of the geometry as possible without loosing too much information, or requiring a lot of computational power.
3. *Computation of similarity, distance, and classical metrics* between the test grid or voxelization and the reference grid (reference deformed volume). Based on related work which details the correlation among different metrics, we employ and report the following: Hausdorff distance, Jaccard index, adjusted Rand index, mutual information, sensitivity, specificity, and precision [24]. These metrics are computed on the entire geometry that represents the liver (surface). The latter has been extracted from the deformed liver volume (output of the PBSM algorithm) as it carries more information about the registration than a partial surface does.

### Experiment 1: orientation

The first experiment focuses on evaluating the effect of orientation by using the aforementioned pipeline. We define orientation as being the direction from which the target surface is available. For consistency, the parameters are fixed across different target surfaces of a data set. As a soft tissue deformation is nonlinear, partial surfaces bisected in different planes may better describe the deformation and help identify the relevant orientations. The experimental design relies on this idea.

For each data set, the full target surface is bisected into six surfaces: six different partial target surfaces, i.e., *XZ*, *XZ*-hemi, *YZ*, *YZ*-hemi, *XY*, *XY*-hemi. Each partial target surface is a bisection of the surface at the center of mass (e.g., *XZ* is bisected in the *xz*-plane, normal to the *Y*-axis). The hemi-surfaces are surfaces that are bisected in a second iteration in the same direction of the normal, as shown in Fig. 2. Then, we run the evaluation pipeline for each data set (i.e., in silico and phantom) and per target surface.

**Table 1:** Surface area (%) of each considered partial target surface

	*XZ*	*XZ*-hemi	*YZ*	*YZ*-hemi	*XY*	*XY*-hemi
in silico	57.5	26.3	52.3	19.3	55.3	20.8
Phantom	59.6	32.2	48.6	24.7	34.5	19.0

Each row reports a data set employed in the experiments. Each column reports the type of bisection used to create the target surface (full, *XZ*, etc.)

In the experimental phantom data set, the two partial surfaces from CT and from endoscopic stereo images have a surface area of 21% and $\sim$ 20%, respectively. Since the liver in each data set is different, the aforementioned bisections result in different target surface areas, as reported in Table 1.

**Fig. 3:**
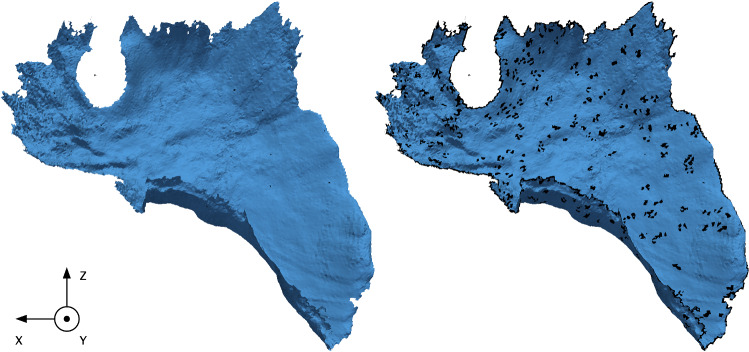
Experiment 2. Visual comparison of each post-processed surface to the original stitched mesh. The output surface meshes are shown after applying strategy (a) on the left, and after applying strategy (b) on the right, respectively. Each superimposes the original stitched mesh (black). Strategy (b) changes mesh elements of the target surface the most, as more elements are visible after filtering (black)

### Experiment 2: mesh quality

The second experiment focuses on evaluating the effect of non-manifold geometry and noise on registration performance by using two automatic post-processing strategies. Experimentally, there are a number of things that can go wrong when creating or importing a surface or mesh (e.g., non-manifold geometry). Non-manifold geometry is defined as geometry that cannot exist in the real world; some examples are an edge is incident to more than two faces, adjacent faces with normals pointing in opposite directions, and two or more faces that are connected only by a vertex and not by an edge. Indeed, it can be difficult to fix such occurrences by simply viewing them. The rational is to use a realistic case, or a geometry created experimentally. Hence, we use the stitched intra-operative surface from the phantom data set.

To address the aforementioned surface mesh problems, we define two strategies: Strategy (a) does a quick pass on the surface mesh without drastically changing the geometry, while strategy (b) removes all non-manifold geometry and fills in any consequent holes by relying on local geometry.

Strategy (a) comprises three consecutive removal steps: (1) duplicate vertices, (2) disconnect vertices and edges, and (3) separate mesh(es). While strategy (b) comprises seven consecutive steps: (1) remove duplicate vertices, (2) remove zero area faces and zero length edges, (3) make all faces convex, (4) remove all non-manifold vertices and edges, (5) fill in holes using boundary edge loops [28], (6) triangulate faces, and (7) remove disconnected vertices and edges.

We define mesh elements as vertices, faces, and edges. Each resulting mesh is used as target surface for registration. Results are evaluated using the aforementioned evaluation pipeline (c.f. experiment 1).

**Table 2:** Registration results for the in silico data set

Metric	Full	*XZ*	*XZ*-hemi	*YZ*	*YZ*-hemi	*XY*	*XY*-hemi
JACRD	0.96	0.91	0.91	0.94	0.74	0.93	0.74
ADJRIND	0.95	0.94	0.88	0.93	0.65	0.91	0.65
MTLINFO	0.84	0.78	0.71	0.75	0.46	0.73	0.46
RMSE	77.16	77.54	77.20	77.73	75.82	77.71	75.82

Each row reports a metric, by descending order: Jaccard, adjusted Rand index, mutual information, and the RMSE. Each column corresponds to one registration, that is to say the registration of the volume to the given target surface (e.g., Full, *XZ*, etc.). The RMSE is computed using the reference deformed surface that is provided in the in silico data set

**Table 3:** Registration results for the experimental phantom data

Metric	Full	*XZ*	*XZ*-hemi	*YZ*	*YZ*-hemi	*XY*	*XY*-hemi	CT$^\mathrm{a}$	Endo$^\mathrm{a}$
JACRD	0.93	0.93	0.93	0.83	0.79	0.87	0.83	0.87	0.80
ADJRIND	0.92	0.92	0.92	0.8	0.75	0.84	0.8	0.85	0.76
MTLINFO	0.72	0.7	0.57	0.52	0.62	0.57	0.45	0.62	0.54
RMSE$^\mathrm{b}$	0	6.14	6.14	11.71	5.56	2.47	7.34	2.52	3.76

Each row reports a metric, by descending order: Jaccard, adjusted Rand index, mutual information, and the RMSE. Each column corresponds to one registration using the PBSM algorithm, that is to say the registration of the volume to the given target surface (e.g., Full). $^\mathrm{a}$ CT and Endo are partial surfaces obtained from the CT scan and the endoscope, respectively. $^\mathrm{b}$ The RMSE is computed by using each resulting deformed surface. As the full surface from CT is the reference, the RMSE cannot be calculated due to the lack of correspondence between mesh vertices, i.e., different numbers of vertices and vertex indices. It is possible to decimate the surface to decrease the number of vertices, yet the correspondence of vertex indices cannot be done. In turn, we use the full deformed surface as reference to calculate the RMSE. This is yet another reason why we opted for a uniform voxel grid to compute relevant registration metrics

**Table 4:** Hausdorff distance (in voxels) for each volume-to-surface registration

	Full	*XZ*	*XZ*-hemi	*YZ*	*YZ*-hemi	*XY*	*XY*-hemi
in silico	22.0	33.5	88.6	30.0	170.6	36.9	170.6
Phantom	106.4	109.8	109.8	112.2	119.5	115.5	106.8

The distance was computed to the voxelized deformed reference volume. Each cell of the table corresponds to one registration. Each column reports the target surface employed by the PBSM method. The baseline (i.e., using the initial and non-deformed surface) results in 170.60 and 181.86 for the in silico and experimental phantom data sets, respectively. The use of the initial surface makes it possible to establish what it means not to use a registration and to have no deformation. Additional distance values are 115.2 voxels for the partial CT scan and 155.01 voxels for the stitched surface or Endo

### Experiment 3: surface area size

The third and last experiments focus on evaluating the effect of surface mesh size provided that the target mesh has a good quality.

As good quality refers to the absence of noise and non-manifold geometry, we choose the single manifold target surface obtained in experiment 2 (strategy b). Then, it is bisected along the *Y*-axis direction in a *s* stepwise increment with $s=5$; the latter is arbitrarily chosen in the local coordinate system. This bisection is carried out in the same direction of the indentation experiment and results in twelve target surfaces. The rational behind using the *Y*-axis as a direction for bisecting the meshes is supported by the accessibility of the left liver lobe. Moreover, it is a coincidence that most of the non-rigid deformation is exerted along the same axis (Fig. 3).

As in experiment 2, each resulting surface is used for registration and results are evaluated. The surface area size is reported in Table 6.

## Results

We report below results for each experiment.

### Experiment 1: orientation

For the first experiment, we report the results of our analysis in Tables 2, 3, and 4. In the in silico data set, we conducted an ANOVA to investigate the interaction effect of target surface size on the Hausdorff distance (in voxel as reported in Table 4). We found a significant difference when using different target surface sizes, as the null hypothesis was rejected with a *p* value or $P=2e{-16}$.

In Table 2, we report registration results for the different target surfaces shown in Fig. 1. The term *significance* is only employed when the *p* value *P*, such as $P<5e-2$. This permits us to formulate the hypothesis that information enclosed in these partial target surfaces is enough to match the nonlinear deformation, with a surface area size of 52.3 and 55.3%, respectively. Our hypothesis is confirmed by the low range of values for the Hausdorff distance of partial surfaces *YZ* and *XY*, 30.0 voxels and 36.9 voxels, respectively (Table 4).

For the experimental phantom data set, we observed larger values for the Hausdorff distance. Compared to the in silico data set, the distance values were larger by a factor of 4.7 when using the full target surface and reached a factor of 7 for the stitched partial surface. We conducted a one sample *t* test for these distances to find whether they are significantly different from the mean value of 106.44 voxels, when using a full surface. As the null hypothesis was not rejected with a *p* value of 0.073 ($P<5e-2$), we concluded that the different registration results are not significantly different from results for the phantom data set.

Moreover, we calculated *P* of the different Pearson’s product–moment correlation. In both data sets, we found that the *XZ* and the *XZ*-hemi-surfaces were on par with the full surface results. On the contrary, we found that that there was no difference between using *XY*, *YZ*, *XY*-hemi, *XY*-hemi, or the partial surface from CT. This uncovered the importance of orientation, in this particular case the *Y*-axis orientation.

We concluded that the target surface size impacted registration results and that results were on par in terms of orientation, across both data sets.

### Experiment 2: mesh quality

For the second experiment, motivated by the fact that intra-operative mesh creation does not always produce good results and little to no work has been in the domain application to improve the created geometry, we investigated the effect of bad mesh quality on registration results. In the scope of this manuscript, this specifically relates to noisy and non-manifold meshes, which are used as input for the task of registering a volume to a target surface. As reported in Table 5, the evaluation shows a clear improvement in the registration performance by using post-processing strategies, especially strategy (b).

**Table 5:** Results for each post-processing strategy (a) and (b) compared to the original Endo target surface (no strategy or post-processing)

Metrics/Strategy	none (Endo)	(a)	(b)
Surface area (%)	$\sim$ 20	19.8	18.5
JACRD	0.80	0.81	0.87
ADJRIND	0.76	0.78	0.84
MTLINFO	0.54	0.55	0.61
PRCISON	0.85	0.86	0.93
RMSE	3.76	2.90	6.87

Each row reports a metric, by descending order: Jaccard, adjusted Rand index, mutual information, and the RMSE. Each column reports the target surface used in the registration experiment. Compared to the other metrics computed on the voxelized volume of each deformed liver, the RMSE is calculated using surface meshes with the Stanford triangle format (STL). The RMSE is computed and compared to the full surface

**Table 6:** Surface area size (%) of each of the twelve bisections along the *Y*-axis

1	2	3	4	5	6	7	8	9	10	11	12
1.58	3.83	6.65	8.64	10.36	12.37	14.39	16.54	17.65	18.21	18.47	18.5

**Fig. 4:**
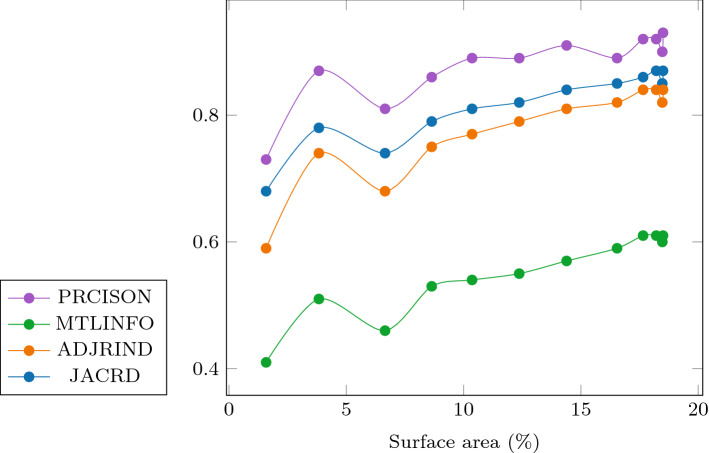
Quantitative metrics as a function of the intra-operative surface area (in percentage). The reported metrics are Jaccard coefficient, adjusted Rand index, mutual information, and precision. The more the surface area increases, the higher each metric reported is. All reported metrics show a similar and correlating trend when the surface size increases

Between strategies (a) and (b), we observe a decrease in surface area size of 0.2% and 1.5% due to the removal of noisy and non-manifold mesh elements. However, thanks to the removal of unrealistic geometry, the registration performance improved across all metrics. This is valid except for the RMSE for strategy (b). Indeed, such an increase in the RMSE may be explained by the loss of too many vertices, faces, and edges compared to the original geometry. Indeed, the RMSE metric might not be suitable to properly account for improvement in registration results, as it only accounts for surface-to-surface changes and may be too sensitive to changes introduced by strategy (b). Moreover, thanks to the removal of all non-manifold geometry, we observed an overall improvement for strategy (b). That is to say, an improvement in each metric: Jaccard by 6.9%, the adjusted Rand index by 7.1%, the mutual information by 9.8%, and the precision by 7.5%.

### Experiment 3: surface size

For the third and last experiments, motivated by knowing how much surface can be used for acceptable registration results we have iteratively bisected the Endo surface along one axis. In this experiment, this corresponds to the direction in which the indentation experiment was carried out: The *Y*-axis. Indeed, this corresponds to a sensitivity analysis as we investigate the quantitative registration metrics for each of the twelve bisections (Table 6). This corresponds to twelve registrations using each of the resulting surfaces as target surface for the registration.

**Fig. 5:**
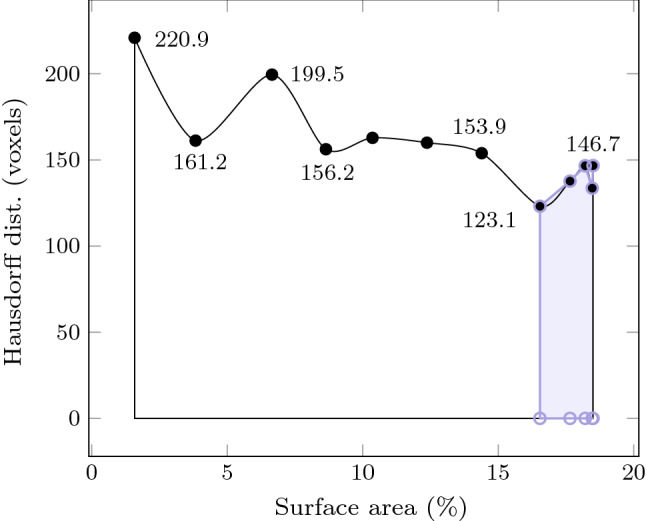
The Hausdorff distance (in voxels) as a function of the available intra-operative surface area (in percentage). As the surface area increases, the better the registration, hence the smaller the distance. The interval of values with acceptable registration results is highlighted in blue

**Fig. 6:**
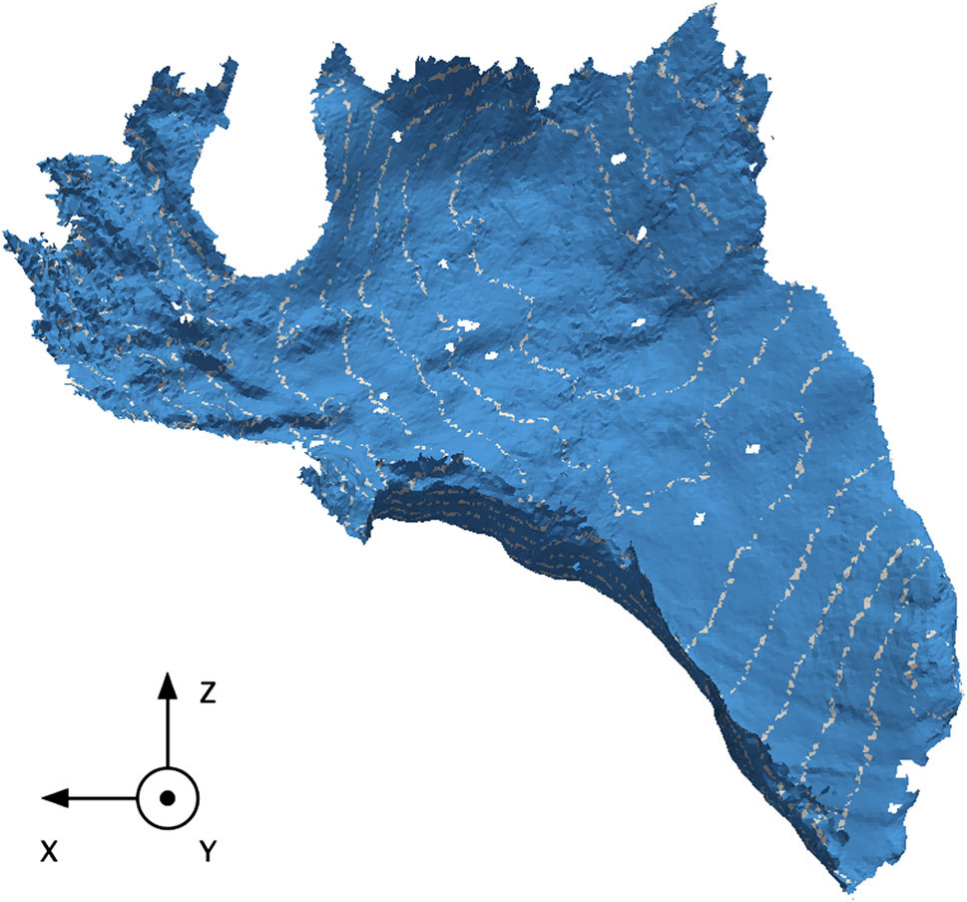
Experiment 3. Illustration of the bisections in the *Y*-axis (indentation direction). Target surface mesh from strategy (b) shown in blue, while the extent of each bisection is shown in white

In Fig. 4, we observe a general trend that shows an increase in registration metrics with surface size. As 20% of the liver organ surface was captured, a particular interest was to find the minimum required surface size to achieve acceptable registration results [22]. To do so, we reported the Hausdorff distance for each bisection in Fig. 5. We found out that, when the surface is in the deformation direction, a target surface size of 16.54% (bisection 8) was satisfactory to achieve good registration results. In this instance, the deformation direction refers to the direction in which the indentation experiment was carried out, that is to say the *Y*-axis as shown in Fig. 6.

## Discussion

First, our findings indicated that an intra-operative surface with an area of less than 20% is challenging to register, especially if the available surface is not oriented in the deformation direction. By comparing registration results from using different target surfaces for the phantom data set, we found that the *XZ* and *XZ*-hemi-surfaces achieved results that are on par with the full surface. We hypothesize that these target surfaces contain enough information about the deformation. Although the *XZ*-hemi surface covered 26.3% (in silico) and 32.3% (phantom) of the full target surface, results indicated that it achieved a good registration performance across both data sets. These results pointed at the relevancy of orientation as both data sets have a similar general direction for the forces exerted on the liver and showed that it is important to obtain a target surface in the general direction of the deformation. However, our findings also showed that mesh quality greatly impacts the registration performance. In our case, and once the target surface mesh quality was improved, only 16.5% of the target surface mesh was necessary in our experiment. We speculate that this also depends on the deformation and its extent, and possibly the organ of interest. Previous work that employs a different spatial discretization, particularly a voxel representation, has found that a target surface of $\sim$ 20% of the visible organ surface was necessary and corresponded to the largest drop in error [15]. Indeed, this corresponds to the size of the unfiltered stitched surface (Endo) used in [23] and is roughly half the amount of accessible liver surface that is accessible. Indeed, in laparoscopic liver surgery, up to 40% of the liver surface may be visible. This value may increase by 20 to 30% when the liver is mobilized. However, we argue that improving the mesh quality and acquiring a surface in the correct orientation permits to diminish the required surface size to 16.5%. Moreover, our results show that efforts to obtain a larger surface size should be considered, especially from intra-operative imaging. Indeed, even if the target surface size is increased for a couple of percentages, the registration performance is overall improved; as observed by the general trend in Fig. 4. To achieve this feat, however difficult, a slow and careful panning of the endoscope across the visible and accessible organ’s surface is preferable. Our hypothesis is that by doing so, a better image sequence could result, in turn improving the stitching and the reconstruction of the target surface.

Second, in an effort to be comparable with existing and more general state-of-the-art methods, the mean geometrical error was calculated for both the PBSM method and the coherent point drift (CPD) algorithm using a full target surface [12]. Results showed a higher accuracy in the case of the PBSM method by a factor of 4. We found a mean error of 0.48 mm, as opposed to 1.96 mm for the CPD algorithm for the in silico data set. These values corroborate with previous research contrasting these methods in terms of displacement error and accuracy, respectively  [22].

Third, our findings point toward the need for research efforts in the usage and evaluation of partial surfaces in non-rigid registration schemes. Their usage is challenging, especially when the employed surface lacks salient features and contains errors or even noise (especially in the case of a stitched surface). Further endeavors in the operating room to acquire intra-operative target surfaces for registration are necessary. Future efforts could focus on smoothing such surfaces, or introducing a uniform noise to characterize quantitatively the influence of noise, or even validating our findings in the case of other organs (e.g., the kidney).

Fourth, the herein presented evaluation pipeline is generalizable. However, this pipeline is computationally intensive and time intensive as the voxel grid size is large. In future work, it is relevant to conduct a sensitivity analysis to investigate both the grid size and its effects on the reported metrics. In practice, this would enable us to use the minimum required grid size to efficiently evaluate registration results, potentially in a clinical setting.

Fifth, our evaluation framework was motivated by different facts: (a) Target surface meshes and volumes may differ in size and geometry (number of nodes, edges). This makes a comparison difficult using meshes or volumes of different resolutions, (b) the voxelized geometry cannot be simply converted to millimeters in case of anisotropic voxel sizes, which is why we propose a $1024^3$ grid that carries the same dimension or a uniform resolution size, and (c) a convergence analysis is not always possible, especially in a clinical setting. This is particularly true when the intra-operative stitched surface does not geometrically match the preoperative geometry (i.e., edges and nodes represent different physical parts of the real world/in this case liver organ). Indeed, this was the case for the stitched intra-operative target surface and the preoperative geometry.

Sixth, the hole filling step in strategy (b) relies on existing information, that is to say local and neighboring geometry. Indeed, since the reconstructed geometry relies on the neighboring topology, it is advisable that future work integrates such a step with care. As the size of the holes is larger, the larger the uncertainty for the boundary edge loops filling algorithm. Hence, it would be important to investigate the threshold at which the size no longer becomes suitable for this algorithm.

Seventh, although experiment 1 is designed with the motivation of investigating orientation, it is a difficult task to achieve. Indeed, we do not know how close the surface orientation has to be to the deformation direction to achieve acceptable registrations. Future work could look at the orientation and its relation to the deformation direction by sampling the space given a user settable deformation direction. That is to say, such a sampling could be carried out in one specific direction to reduce the computational expense of the evaluation and possibly could have an overview for such a characterization by focusing on a couple of relevant metrics.

Eighth, there exist initial liver registration methods which use only the intra-operative endoscopic image feed. Indeed, such methods do not rely on the representation of an object of interest, that is to say its geometry. Some of these methods rely on feature extraction, while others combine feature extraction with feature tracking (i.e., mosaicing). These approaches fit preoperatively extracted features directly into the intra-operative image data  [1, 7, 27]. Although these approaches are different from the presented non-rigid volume-to-surface registration method we evaluate, we hypothesize that these approaches may benefit from sensitivity analyses that entail varying the signal-to-noise ratio, image resolution, and other limiting factors that may influence the registration performance.

Ninth, and in a clinical setting, the orientation can be approximated. However important, the orientation in which the liver is, is difficult to grasp during surgery. Our findings point toward the importance of such a factor only when a small surface area is accessible. Provided a panning shot is accomplished, orientation is still important yet granted secondary to the problem. It becomes important only when the surface does not carry enough information of the deformation. Logically, we may think of surface size; however, the order of the tetrahedra that define the surface mesh affects this result. This has been shown in silico in the thesis of S. Suwelack that the second-order tetrahedra better carry the deformation than the first-order tetrahedra. We foresee that future work may better study and characterize such a point in a realistic setting.

Tenth and last, we designed different experiments in an effort to improve registration results. Results from our case study prompt for further investigations that would integrate more observations or data sets.

## Conclusion

As a means of conclusion, we bring forth three points. First, the evaluation pipeline permitted us to investigate the impact of target surface size on registration performance across two data sets. By employing directional bisections, we found that different orientations, hence parts of the full target surface, contain different deformation information. This characterization plays a pivotal role and shows the variability of the information carried out by a deformation and its impact on registration results.

Second, we found that the unfiltered intra-operative or stitched surface, with approximately 20% of surface area size, was of good size to achieve acceptable registration results. Yet, in this particular case and compared to the full surface, we observed a loss of 10.5%, and 7.6% in precision when compared to the partial surface from CT, respectively (Fig. 2). This was no longer the case after removing noise and non-manifold geometry. We found out that a minimum surface size of 16.5% achieved good results (Fig. 4).

Third and last, mesh quality is of paramount importance. As seen in experiment 2, each post-processing strategy reduced the target surface size, yet improved registration results.

Our findings serve as a standard for future volume-to-surface-based registration methods and could motivate future endeavors in obtaining intra-operative surfaces of better quality and with a larger surface area size.

## Data Availability

All materials employed herein are available under a Creative Commons Attribution NonCommercial ShareAlike 4.0 License at GitHub under EvalPBSM.
